# Awareness, perceived barriers, and facilitators related to premarital screening and genetic counseling among Palestinian adults

**DOI:** 10.3389/fpubh.2026.1853380

**Published:** 2026-07-10

**Authors:** Alhareth M. Amro, Salahaldeen Deeb, Rand Zoghayyer, Jenan Sinokrot, Husam Amarin, Alaa Qasem, Abdallah Dwayat, Malak Manasrah

**Affiliations:** Faculty of Medicine, Al-Quds University, Jerusalem, Palestine

**Keywords:** consanguinity, genetic counseling, genetic diseases, Palestine, premarital screening tests (PMST), public perceptions

## Abstract

**Background:**

Genetic diseases are hereditary conditions caused by DNA abnormalities, and they are an important public health issue worldwide, contributing considerably to morbidity and mortality. Palestine has a high incidence of genetic diseases, attributed to the high percentage of consanguineous marriages. Limited studies have been conducted to determine the level of awareness and knowledge among the Palestinian people about genetic disorders.

**Objective:**

This study assessed awareness, perceived barriers, and facilitators related to premarital screening tests (PMST), genetic testing, and genetic counseling among Palestinian adults recruited through a web-based survey.

**Methods:**

A cross-sectional, web-based survey was conducted in Palestine between August 2025 and January 2026 using an online, structured, self-administered questionnaire. Eligible participants were Palestinian residents aged ≥18 years who were able to complete the questionnaire online. Three submissions selecting “Under 18” were excluded before analysis, leaving 419 adult respondents. Descriptive statistics, bivariate tests, reliability analysis, and multivariable linear and logistic regression models were performed. Because the survey link was distributed through convenience and snowball sampling, a conventional response rate could not be calculated.

**Results:**

Among 419 adult respondents, 98.8% had heard of genetic diseases, 63.7% had heard of genetic counseling, and only 8.8% had visited a genetic diseases clinic. The mean BarrierScore was 4.65 ± 1.60, the mean MotivationScore was 6.77 ± 1.40, and the mean TotalScore was 11.42 ± 2.14. Using descriptive thresholds, 218 respondents (52.0%) had a high TotalScore, 185 (44.2%) had a moderate TotalScore, and 16 (3.8%) had a low TotalScore. In multivariable linear regression, higher TotalScore was independently associated with family history of genetic disease, correct knowledge that genetic diseases can appear at any stage of life, having heard of genetic counseling, knowing that PMST does not cover all genetic diseases, and stronger agreement that PMST is important.

**Conclusion:**

In this convenience sample of Palestinian adults, general awareness of genetic diseases was high, but awareness and use of genetic counseling remained incomplete. The findings should not be interpreted as nationally representative. They support the need for culturally sensitive education about PMST limitations, wider availability of genetic counseling, and future representative research.

## Introduction

Genetic diseases are inherited disorders resulting from abnormalities in DNA that impose substantial health, social, and economic burdens on affected individuals and their families (1). Worldwide, genetic disorders constitute a major public health concern, as they significantly contribute to morbidity and mortality, particularly during early life (2). Similar to many Middle Eastern countries, Palestine has a high prevalence of hereditary and genetic disorders, largely due to cultural practices and demographic characteristics, most notably the high rate of consanguineous marriages, which increases the likelihood of inheriting deleterious genes from both parents (3).

Despite the considerable health impact of genetic disorders and the proven benefits of preventive strategies, limited research has explored the extent of awareness and knowledge among the general Palestinian population regarding genetic diseases, their causes, and the available preventive and diagnostic tools (4). Public knowledge in this area is crucial, as it directly influences attitudes toward genetic testing, counseling, and other preventive health behaviors that can ultimately reduce disease burden (5). Therefore, understanding the level of awareness and knowledge within the Palestinian community is essential for identifying misconceptions and knowledge gaps that should be addressed through targeted health education programs (6).

Limited research has examined awareness of PMST limitations and genetic counseling among Palestinian adults outside specialized clinical settings. Understanding awareness, perceived barriers, and facilitators in an online community sample can help identify practical gaps that should be addressed in future public health programs. This study assessed awareness, perceived barriers, and facilitators related to PMST, genetic testing, and genetic counseling among Palestinian adults recruited through a web-based survey, and explored factors associated with more favorable composite scores.

## Methodology

### Study design and setting

A cross-sectional, web-based survey was conducted in Palestine to assess awareness and perceptions regarding genetic diseases, premarital screening tests (PMST), genetic testing, and genetic counseling. Data were collected using a structured, self-administered Google Forms questionnaire between August 2025 and January 2026. The survey was disseminated electronically through commonly used online platforms and participant sharing; therefore, recruitment followed convenience and snowball sampling rather than probability sampling.

The online format enabled broad geographic reach and rapid recruitment, but it also introduced important selection bias. Individuals who were younger, more educated, more interested in health topics, or more digitally connected were more likely to encounter and complete the survey. The results are therefore interpreted as describing this web-based convenience sample and should not be generalized to all Palestinian adults.

Because this was an internet-based survey, reporting was strengthened by adding items aligned with the Checklist for Reporting Results of Internet E-Surveys (CHERRIES) (7). The survey link was distributed openly and could be forwarded by participants. The number of people who viewed the invitation or opened the survey link was not recorded. Consequently, view rate, participation rate, completion rate, and conventional response rate could not be calculated.

### Participants

Eligible participants were Palestinian residents aged ≥18 years who were able to read and complete the questionnaire online. Individuals who did not meet the age or residency criteria, or who submitted incomplete responses for core outcome variables, were excluded. During data cleaning, three submissions selecting “Under 18” were removed to maintain the adult eligibility criterion. The final analytic sample included 419 adult respondents.

### Questionnaire development and cultural adaptation

The questionnaire was adapted from the published instrument by Al Eissa et al., which assessed perceptions of genetic diseases and PMST-related attitudes and decision drivers in Saudi Arabia (8). The source article was published as an open-access article under a Creative Commons Attribution 4.0 International License, which permits use and adaptation with appropriate attribution, a link to the license, and indication of changes. Therefore, separate written permission was not required, provided that the original source is cited and the adaptation is clearly acknowledged.

The adapted sections included sociodemographic characteristics; awareness of genetic diseases and genetic counseling; personal and family history of hereditary disease; prior genetic testing; awareness of PMST coverage and limitations; attitudes toward PMST importance; awareness of preimplantation genetic testing/diagnosis (PGT/PGD); willingness to request genetic testing or genetic counseling; and perceived barriers and facilitators influencing genetic testing decisions. These domains correspond to the public-opinion and decision-driver sections of the original questionnaire.

Items were modified for the Palestinian context by adapting governorate/region options, simplifying partner-kinship categories, aligning terminology with PMST and counseling services as understood locally, and removing Saudi-specific items such as tribe-specific marriage categories, Saudi program identifiers, and Saudi health-system references that were not applicable to Palestine. These modifications were made to improve local relevance and reduce respondent burden while preserving the main constructs of the original instrument.

A separate formal pilot study was not conducted after adaptation. Before dissemination, the research team reviewed the Arabic wording, cultural appropriateness, logical flow, and clarity of the online form. The absence of a formal pilot validation study is acknowledged as a limitation. In the present dataset, reliability was examined using KR-20/Cronbach’s alpha because the scored items were binary.

### Study variables and measures

Sociodemographic variables included age, sex, educational level, marital status, employment, income, region of residence, and partner consanguinity status. Exposure and experience variables included having heard of genetic diseases, having heard of genetic counseling, prior attendance at a genetic diseases clinic, personal hereditary disease, family history of genetic disease, prior genetic testing, awareness of PMST limitations, awareness of PGT/PGD, perceived PMST importance, and satisfaction with available information.

The questionnaire also captured perceived barriers and facilitators. Barrier-related items reflected knowledge gaps, uncertainty about personal or family genetic status, misconceptions that PMST covers all genetic diseases, perceived lack of risk to future children, and fear of societal views. Facilitator-related items included affordability, insurance coverage, government systems, awareness campaigns, sharing experiences, maintaining offspring health, reproductive alternatives, and marriage customs.

### Scoring system and outcome classification

Fifteen perception-related items were coded as binary favorable-response indicators. Seven items formed the BarrierScore (0–7), where a value of 1 indicated lower perceived barrier or more accurate knowledge for that item. Eight items formed the MotivationScore (0–8), where a value of 1 indicated endorsement of a facilitator. The TotalScore (0–15) was calculated by summing BarrierScore and MotivationScore.

Because the TotalScore combines conceptually different domains, it was treated as a pragmatic descriptive index of favorable orientation toward informed genetic decision-making rather than a validated unidimensional knowledge scale. BarrierScore and MotivationScore were therefore reported separately, and item-level results were presented to preserve interpretability. TotalScore was also analyzed as a continuous outcome in regression models. For descriptive purposes only, TotalScore was categorized as low (0–7; <50% favorable responses), moderate (8–11; 50 to <80%), or high (12–15; ≥80%). These thresholds were selected on percentage-of-maximum-score grounds and should not be interpreted as externally validated diagnostic cutoffs.

### Data management and statistical analysis

Survey responses were downloaded from Google Forms and screened for eligibility, completeness of core outcomes, and coding consistency. Descriptive statistics were reported as frequencies and percentages for categorical variables and as means with standard deviations for continuous scores. Bivariate comparisons of TotalScore were conducted using Welch’s t-test for two-group comparisons and one-way ANOVA for variables with more than two categories. Effect sizes were reported as Cohen’s d for two-group comparisons and eta-squared (η^2^) for ANOVA. Item-level differences by sex and region were evaluated using chi-square tests with Cramér’s V.

Internal consistency of the binary scored items was assessed using KR-20/Cronbach’s alpha for the TotalScore, BarrierScore, and MotivationScore. Multivariable linear regression was used to identify independent predictors of TotalScore. Logistic regression was used as a secondary descriptive analysis predicting high TotalScore (≥12). Covariates were selected based on conceptual importance and the original analytic plan. Two-sided *p*-values <0.05 were considered statistically significant.

### Ethical approval

The study protocol, questionnaire, and electronic informed-consent procedure were reviewed and approved by the Research Ethics Committee at Al-Quds University (approval no. 409/REC/2025). Participation was anonymous and voluntary. Electronic informed consent was obtained before participants accessed the survey questions. Data were handled confidentially and analyzed in aggregate form, with no personally identifying information used in reporting.

## Result

### Participant characteristics

A total of 419 adult respondents were included in the analysis after exclusion of three under-18 submissions (Table 1). The sample was predominantly female (74.7%), and the most common age group was 22–26 years (42.2%). Educational attainment was high, with 90.0% reporting university education or higher. Married participants represented 52.0% of the sample. Respondents were concentrated in the Central region (61.6%), followed by the South (26.0%) and North (12.4%). Exposure-related variables showed very high awareness of genetic diseases (98.8%), moderate awareness of genetic counseling (63.7%), and low prior attendance at a genetic diseases clinic (8.8%). A family history of genetic disease was reported by 32.2, and 8.8% reported a personal hereditary disease. More than half reported no prior genetic testing (58.9%), while 27.9% reported premarital screening and 10.7% reported other genetic testing.

**Table 1:** Participant characteristics (adult analytic sample, *n* = 419).

Characteristic	Category	n (%)
Sex	Female	313 (74.7%)
	Male	106 (25.3%)
Age group (years)	18–21	31 (7.4%)
	22–26	177 (42.2%)
	27–30	60 (14.3%)
	31–35	37 (8.8%)
	36–45	63 (15.0%)
	Over 45	51 (12.2%)
Education	University+	377 (90.0%)
	High school or less	42 (10.0%)
Marital status	Married	218 (52.0%)
	Single	152 (36.3%)
	Engaged	41 (9.8%)
	Other	8 (1.9%)
Region	Central	258 (61.6%)
	North	52 (12.4%)
	South	109 (26.0%)
Work status	unemployed	118 (28.2%)
	student	83 (19.8%)
	Private sector employee	103 (24.6%)
	government employee	34 (8.1%)
	Self-employed person	28 (6.7%)
	I work part-time	43 (10.3%)
	Retired	10 (2.4%)
Income level	Low income (insufficient for basic needs)	83 (19.8%)
	Average income (enough for basic needs, not luxuries)	269 (64.2%)
	High income (enough for basic needs and luxury)	67 (16.0%)
Consanguinity status	Non-consanguineous	243 (58.0%)
	Consanguineous	58 (13.8%)
	No partner yet	118 (28.2%)
Heard of genetic diseases	yes	414 (98.8%)
	no	5 (1.2%)
Heard of genetic counseling	Yes	267 (63.7%)
	No	152 (36.3%)
Visited genetic diseases clinic	Yes	37 (8.8%)
	No	382 (91.2%)
Family history of genetic disease	Yes	135 (32.2%)
	No	235 (56.1%)
	Do not know	49 (11.7%)
Personal hereditary disease	Yes	37 (8.8%)
	No	382 (91.2%)
Prior genetic testing history	No prior testing	247 (58.9%)
	Premarital screening	117 (27.9%)
	Other genetic testing	45 (10.7%)
	Uncertain	10 (2.4%)

### Composite score distribution, reliability, and score interpretation

**Figure 1:**
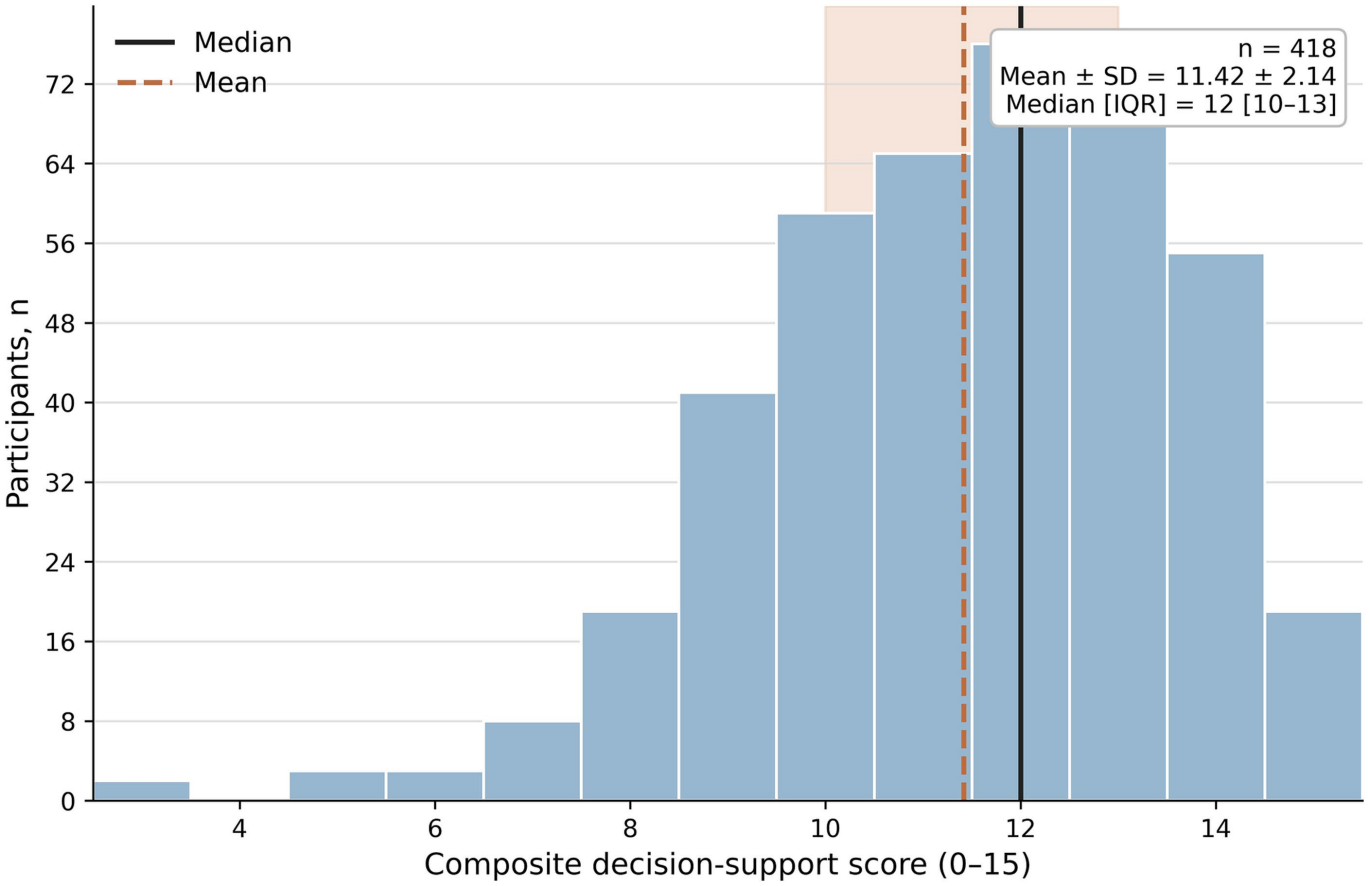
TotalScore by awareness of genetic counseling.

Composite scoring was based on 15 perception-related items, including 7 barrier-oriented items and 8 facilitator-oriented items. The mean BarrierScore was 4.65 ± 1.60, the mean MotivationScore was 6.77 ± 1.40, and the mean TotalScore was 11.42 ± 2.14. Using descriptive thresholds, 218 respondents (52.0%) had high TotalScore, 185 (44.2%) had moderate TotalScore, and 16 (3.8%) had low TotalScore (Figure 1).

Internal consistency was modest for the TotalScore (KR-20/Cronbach’s alpha = 0.49), BarrierScore (alpha = 0.47), and MotivationScore (alpha = 0.60). BarrierScore and MotivationScore were essentially uncorrelated (Pearson *r* = 0.01, *p* = 0.868), supporting the decision to interpret the subscales and item-level findings separately rather than treating TotalScore as a purely unidimensional construct (Table 2).

**Table 2:** Composite score comparisons across key respondent characteristics.

Variable	Category	n	BarrierScore	MotivationScore	TotalScore	*p*	Effect
Sex	Female	313	4.61 ± 1.58	6.86 ± 1.36	11.47 ± 2.13	0.377	d = 0.10
	Male	106	4.75 ± 1.67	6.50 ± 1.48	11.25 ± 2.17		
Age group (years)	18–21	31	3.94 ± 1.44	7.19 ± 1.33	11.13 ± 1.89	0.309	η^2^ = 0.014
	22–26	177	4.89 ± 1.55	6.71 ± 1.40	11.60 ± 2.20		
	27–30	60	4.62 ± 1.80	6.53 ± 1.53	11.15 ± 2.22		
	31–35	37	4.81 ± 1.54	6.92 ± 1.06	11.73 ± 1.64		
	36–45	63	4.29 ± 1.59	6.73 ± 1.56	11.02 ± 2.49		
	Over 45	51	4.61 ± 1.54	6.90 ± 1.25	11.51 ± 1.76		
Education	University+	377	4.72 ± 1.59	6.76 ± 1.40	11.48 ± 2.12	0.068	d = 0.32
	High school or less	42	4.02 ± 1.57	6.79 ± 1.41	10.81 ± 2.23		
Region	Central	258	4.59 ± 1.56	6.92 ± 1.37	11.51 ± 2.09	0.176	η^2^ = 0.008
	North	52	4.35 ± 1.60	6.56 ± 1.45	10.90 ± 2.38		
	South	109	4.94 ± 1.68	6.50 ± 1.41	11.44 ± 2.10		
Marital status	Married	218	4.50 ± 1.64	6.80 ± 1.42	11.30 ± 2.26	0.452	η^2^ = 0.006
	Single	152	4.82 ± 1.55	6.78 ± 1.36	11.60 ± 1.94		
	Engaged	41	4.85 ± 1.62	6.39 ± 1.48	11.24 ± 2.18		
	Other	8	4.50 ± 1.31	7.50 ± 0.93	12.00 ± 2.07		
Work status	unemployed	118	4.60 ± 1.64	6.69 ± 1.50	11.29 ± 2.32	0.026	η^2^ = 0.034
	student	83	4.89 ± 1.53	7.12 ± 1.24	12.01 ± 1.75		
	Private sector employee	103	4.66 ± 1.61	6.50 ± 1.36	11.16 ± 2.23		
	government employee	34	4.32 ± 1.80	6.82 ± 1.62	11.15 ± 2.48		
	Self-employed person	28	4.46 ± 1.43	6.25 ± 1.58	10.71 ± 2.00		
	I work part-time	43	4.70 ± 1.57	7.21 ± 0.94	11.91 ± 1.70		
	Retired	10	4.50 ± 1.72	6.90 ± 1.20	11.40 ± 1.58		
Income level	Low income (insufficient for basic needs)	83	4.45 ± 1.59	6.76 ± 1.58	11.20 ± 2.20	0.584	η^2^ = 0.003
	Average income (enough for basic needs, not luxuries)	269	4.66 ± 1.59	6.82 ± 1.33	11.48 ± 2.12		
	High income (enough for basic needs and luxury)	67	4.85 ± 1.67	6.55 ± 1.42	11.40 ± 2.15		
Consanguinity status	Non-consanguineous	243	4.49 ± 1.60	6.72 ± 1.42	11.21 ± 2.18	0.038	η^2^ = 0.016
	Consanguineous	58	4.69 ± 1.66	6.79 ± 1.41	11.48 ± 2.30		
	No partner yet	118	4.97 ± 1.54	6.85 ± 1.35	11.81 ± 1.90		
Family history of genetic disease	Yes	135	4.84 ± 1.48	6.96 ± 1.16	11.81 ± 1.91	<0.001	η^2^ = 0.050
	No	235	4.74 ± 1.63	6.71 ± 1.49	11.45 ± 2.18		
	Do not know	49	3.67 ± 1.46	6.51 ± 1.54	10.18 ± 2.10		
Heard genetic counseling	Yes	267	5.17 ± 1.44	6.75 ± 1.38	11.92 ± 1.99	<0.001	d = 0.68
	No	152	3.74 ± 1.46	6.80 ± 1.44	10.53 ± 2.11		
Knew PMST does not cover all diseases	Yes	308	5.01 ± 1.49	6.77 ± 1.43	11.79 ± 2.07	<0.001	d = 0.68
	No	111	3.64 ± 1.46	6.75 ± 1.32	10.39 ± 1.99		
Knew PMST covers only two disorders	Yes	263	5.05 ± 1.48	6.73 ± 1.45	11.78 ± 2.06	<0.001	d = 0.47
	No	156	3.97 ± 1.57	6.83 ± 1.30	10.80 ± 2.12		
Heard of PGT/PGD	Yes	277	5.01 ± 1.57	6.80 ± 1.40	11.82 ± 2.11	<0.001	d = 0.57
	No	142	3.94 ± 1.41	6.70 ± 1.41	10.63 ± 1.97		
Correct disease-onset knowledge	Correct	354	4.82 ± 1.60	6.83 ± 1.30	11.65 ± 2.02	<0.001	d = 0.73
	Other/Unknown	65	3.71 ± 1.23	6.43 ± 1.85	10.14 ± 2.31		
Visited genetic clinic	Yes	37	5.19 ± 1.65	7.14 ± 1.00	12.32 ± 2.03	0.007	d = 0.47
	No	382	4.60 ± 1.59	6.73 ± 1.43	11.33 ± 2.13		
Personal hereditary disease	Yes	37	5.03 ± 1.14	7.08 ± 1.16	12.11 ± 1.58	0.010	d = 0.36
	No	382	4.61 ± 1.64	6.74 ± 1.42	11.35 ± 2.17		
Prior testing history	No prior testing	247	4.53 ± 1.63	6.74 ± 1.40	11.28 ± 2.10	0.013	η^2^ = 0.026
	Premarital screening	117	4.74 ± 1.62	6.82 ± 1.49	11.56 ± 2.31		
	Other genetic testing	45	5.20 ± 1.41	6.91 ± 1.06	12.11 ± 1.75		
	Uncertain	10	4.00 ± 0.82	6.00 ± 1.70	10.00 ± 1.63		

### Bivariate associations with composite scores

**Figure 2:**
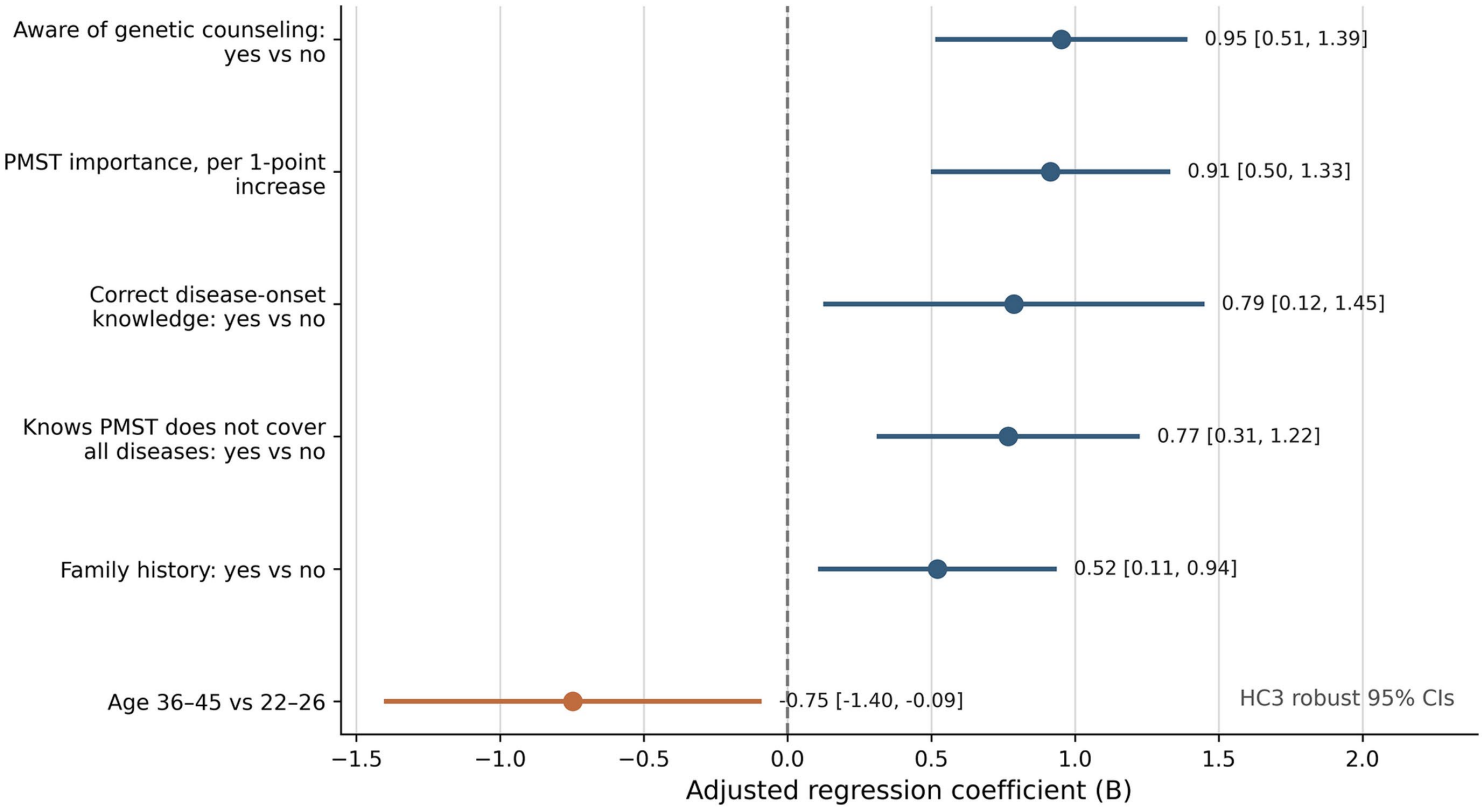
Distribution of TotalScore.

**Figure 3:**
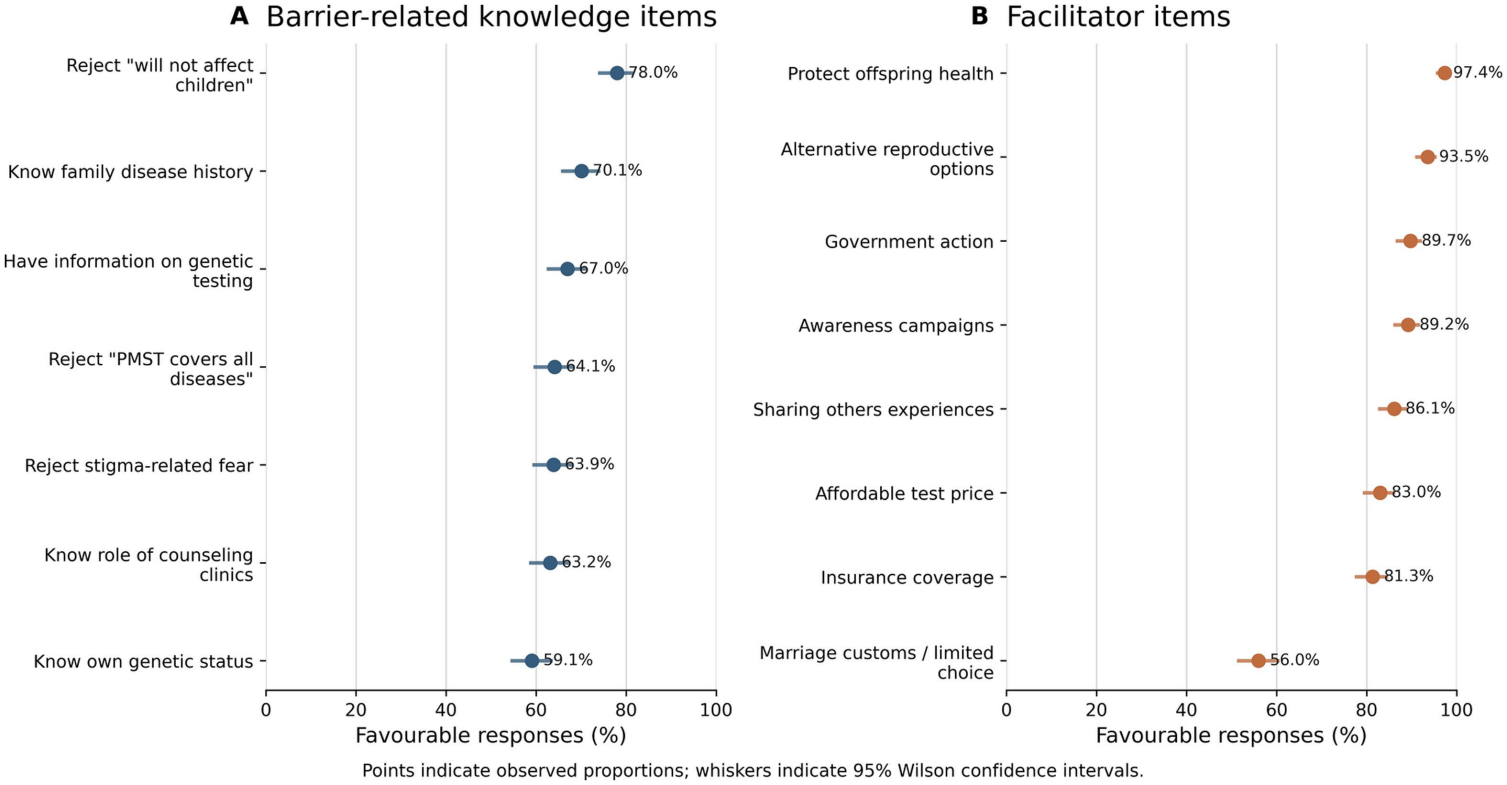
Endorsement of barrier-related knowledge and facilitator items. **(A)** Barrier-related knowledge items. **(B)** Facilitator items. Points indicate observed proportions; whiskers indicate 95% Wilson confidence intervals.

Bivariate comparisons of BarrierScore, MotivationScore, and TotalScore across respondent characteristics are presented in Table 2. TotalScore was higher among respondents with university education or higher than among those with high school education or less, but this difference did not reach conventional statistical significance after adult-only reanalysis (11.48 ± 2.12 vs. 10.81 ± 2.23; *p* = 0.068; d = 0.32). TotalScore differed significantly by family history of genetic disease (*p* < 0.001; η^2^ = 0.050), with the highest mean score among respondents reporting a family history and the lowest among those unsure about family history. Knowledge and exposure variables were consistently associated with higher TotalScore. Respondents who had heard of genetic counseling had higher TotalScore than those who had not (*p* < 0.001; d = 0.68). Knowing that PMST does not cover all genetic diseases (*p* < 0.001; d = 0.68), knowing that PMST covers only two hereditary blood disorders (*p* < 0.001; d = 0.47), awareness of PGT/PGD (*p* < 0.001; d = 0.57), and correct knowledge that genetic diseases can appear at any stage of life (*p* < 0.001; d = 0.73) were also associated with higher TotalScore. Respondents who had visited a genetic diseases clinic had higher TotalScore than those who had not (*p* = 0.007; d = 0.47), and those reporting a personal hereditary disease had higher TotalScore than those who did not (*p* = 0.010; d = 0.36). Prior testing history was also associated with TotalScore (*p* = 0.013; η^2^ = 0.026), with the highest mean score among respondents reporting other genetic testing (Figures 2, 3).

TotalScore did not significantly differ by sex, age group, marital status, income level, or region. Work status and consanguinity status showed statistically significant but small differences in adult-only bivariate analysis; these findings should be interpreted cautiously because of multiple comparisons and uneven subgroup sizes.

### Item-level scoring patterns and subgroup differences

Item-level scoring patterns are summarized in Table 3. Across barrier-oriented items, the highest favorable score was observed for rejecting the statement that genetic disease would not affect future children (78.0%), while the lowest favorable score was for knowing whether one personally has a genetic disease (58.9%). For facilitator items, endorsement was highest for maintaining offspring health (97.4%) and for prior knowledge that testing may contribute to reproductive solutions (93.6%). The least frequently endorsed facilitator was limited marriage choice customs (56.1%).

**Table 3:** Item-level scoring patterns overall and by sex/region with χ^2^ tests.

Domain	Item	Scored as 1 when	Overall (%)	Female (%)	Male (%)	p_Sex	V_Sex	Central (%)	North (%)	South (%)	p_Region	V_Region
Barrier	I know if I have a genetic disease or not	Yes	58.9	55.9	67.9	0.039	0.11	58.5	51.9	63.3	0.380	0.07
Barrier	I know if any member of my family has a genetic disease	Yes	69.9	70.0	69.8	1.000	0.00	68.6	69.2	73.4	0.654	0.05
Barrier	I have information about genetic testing	Yes	66.8	67.1	66.0	0.936	0.01	67.8	57.7	68.8	0.322	0.07
Barrier	I know the role of genetic counseling clinics	Yes	63.0	64.5	58.5	0.318	0.05	64.7	57.7	61.5	0.586	0.05
Barrier	I do not think it will affect my children in the future	No	78.0	77.3	80.2	0.630	0.03	74.0	73.1	89.9	0.002	0.17
Barrier	I believed that premarital screening covered all genetic diseases and would protect my family from them	No	64.2	63.9	65.1	0.916	0.01	62.4	61.5	69.7	0.373	0.07
Barrier	I am afraid of how society will view me if I have a genetic disease and of its future impact on my children	No	64.0	62.6	67.9	0.386	0.05	62.8	63.5	67.0	0.745	0.04
Facilitator	Price of the test is reasonable and affordable	Yes	83.1	83.1	83.0	1.000	0.00	81.4	78.8	89.0	0.143	0.10
Facilitator	Government systems to reduce the spread of these genetic diseases	Yes	89.7	91.7	84.0	0.037	0.11	91.5	94.2	83.5	0.037	0.13
Facilitator	Maintaining the health of offspring	Yes	97.4	97.8	96.2	0.614	0.04	98.4	98.1	94.5	0.091	0.11
Facilitator	Limited choice in marriage/customs imposing marriage between relatives or within the same family	Yes	56.1	58.1	50.0	0.178	0.07	58.5	53.8	51.4	0.425	0.06
Facilitator	Medical insurance covers these tests	Yes	81.4	81.8	80.2	0.825	0.02	82.9	73.1	81.7	0.248	0.08
Facilitator	Awareness campaigns increase knowledge about these diseases	Yes	89.3	91.1	84.0	0.063	0.10	93.4	88.5	79.8	<0.001	0.19
Facilitator	Sharing experiences with others	Yes	86.2	88.5	79.2	0.026	0.12	89.9	86.5	77.1	0.005	0.16
Facilitator	Prior knowledge that testing contributes to reproductive solutions without inheriting these diseases	Yes	93.6	93.6	93.4	1.000	0.00	95.7	82.7	93.6	0.002	0.17

Selected item-level subgroup differences were observed. Males more often reported knowing whether they personally have a genetic disease (67.9% vs. 55.9%; *p* = 0.039; Cramér’s V = 0.11). Females more often endorsed government systems as encouraging genetic testing (91.7% vs. 84.0%; *p* = 0.037; V = 0.11) and sharing experiences with others (88.5% vs. 79.2%; *p* = 0.026; V = 0.12). By region, favorable scoring differed for rejecting the belief that genetic disease would not affect future children (*p* = 0.002; V = 0.17), endorsing government systems (*p* = 0.037; V = 0.13), awareness campaigns (*p* < 0.001; V = 0.19), sharing experiences (*p* = 0.005; V = 0.16), and prior knowledge about reproductive solutions (*p* = 0.002; V = 0.17).

### Multivariable regression models

Multivariable regression results are presented in Table 4. In linear regression, reporting a family history of genetic disease was independently associated with higher TotalScore (B = 0.51, 95% CI 0.10 to 0.92; *p* = 0.015). Correct knowledge that genetic diseases can appear at any stage of life was associated with higher TotalScore (B = 0.73, 95% CI 0.20 to 1.27; *p* = 0.008). Having heard of genetic counseling remained independently associated with higher TotalScore (B = 0.85, 95% CI 0.42 to 1.28; *p* < 0.001), as did knowing that PMST does not cover all genetic diseases (B = 0.65, 95% CI 0.18 to 1.12; *p* = 0.007). Stronger agreement that PMST is important was also positively associated with TotalScore (B = 0.85 per 1-point increase, 95% CI 0.44 to 1.26; *p* < 0.001). Age 36–45 years was associated with lower TotalScore compared with the 22–26-year reference group (B = −0.74, 95% CI − 1.33 to −0.14; *p* = 0.015).

**Table 4:** Multivariable regression results.

Predictor	Linear B (95% CI)	p (Linear)	Logistic OR (95% CI)	*p* (Logistic)
Age: 18–21 (vs 22–26)	0.03 [−0.71, 0.76]	0.944	1.24 [0.51, 2.98]	0.634
Age: 27–30 (vs 22–26)	−0.17 [−0.73, 0.39]	0.547	0.86 [0.44, 1.68]	0.663
Age: 31–35 (vs 22–26)	0.34 [−0.36, 1.03]	0.341	0.87 [0.38, 2.00]	0.745
Age: 36–45 (vs 22–26)	−0.74 [−1.33, −0.14]	0.015	0.57 [0.28, 1.14]	0.109
Age: Over 45 (vs 22–26)	0.11 [−0.50, 0.72]	0.723	1.04 [0.50, 2.18]	0.916
Education: University+ (vs ≤ High school)	0.10 [−0.54, 0.74]	0.756	1.20 [0.54, 2.65]	0.650
Family history: Do not know (vs No)	−0.52 [−1.13, 0.09]	0.095	0.65 [0.30, 1.38]	0.259
Family history: Yes (vs No)	0.51 [0.10, 0.92]	0.015	1.82 [1.11, 2.97]	0.017
Region: North (vs Central)	−0.33 [−0.93, 0.26]	0.274	0.85 [0.42, 1.74]	0.663
Region: South (vs Central)	−0.35 [−0.79, 0.10]	0.125	0.63 [0.37, 1.08]	0.093
Sex: Female (vs Male)	−0.10 [−0.54, 0.34]	0.656	0.97 [0.57, 1.64]	0.911
Correct knowledge: onset can be any life stage (Yes vs. No)	0.73 [0.20, 1.27]	0.008	1.47 [0.75, 2.87]	0.261
Heard of genetic counseling (Yes vs. No)	0.85 [0.42, 1.28]	<0.001	2.76 [1.69, 4.51]	<0.001
Heard of PGT/PGD (Yes vs. No)	0.36 [−0.07, 0.79]	0.103	1.57 [0.95, 2.59]	0.078
Knew PMST does not cover all diseases (Yes vs. No)	0.65 [0.18, 1.12]	0.007	2.49 [1.44, 4.31]	0.001
PMST importance (per 1-point)	0.85 [0.44, 1.26]	<0.001	2.25 [1.33, 3.81]	0.002
Information satisfaction (per 1-point)	0.08 [−0.15, 0.31]	0.489	—	—

In logistic regression predicting high TotalScore (≥12), higher odds were observed among respondents who had heard of genetic counseling (OR = 2.76, 95% CI 1.69 to 4.51; *p* < 0.001), those who knew PMST does not cover all genetic diseases (OR = 2.49, 95% CI 1.44 to 4.31; *p* = 0.001), those reporting a family history of genetic disease (OR = 1.82, 95% CI 1.11 to 2.97; *p* = 0.017), and those with stronger agreement that PMST is important (OR = 2.25 per 1-point increase, 95% CI 1.33 to 3.81; *p* = 0.002).

## Discussion

This study provides evidence on awareness, perceived barriers, and facilitators related to PMST, genetic testing, and genetic counseling among Palestinian adults recruited through an online convenience sample. The findings should be interpreted as sample-specific rather than nationally representative. Within this sample, almost all respondents had heard of genetic diseases, but awareness and use of genetic counseling were more limited. Only 63.7% had heard of genetic counseling, and only 8.8% had ever visited a genetic diseases clinic. This gap between general awareness and service use is important because PMST alone may not address the full range of hereditary risks relevant to couples and families.

The sample was highly educated, predominantly female, and recruited entirely online. These characteristics likely inflated awareness estimates and limit generalizability. Consequently, statements about awareness should refer to “respondents” or “participants in this sample” rather than to Palestinian adults as a whole. The high proportion of university-educated respondents may also explain why several knowledge-related indicators were relatively favorable compared with what might be expected in a nationally representative sample including older, rural, less educated, and digitally marginalized groups.

Near-universal awareness of genetic diseases likely reflects the high regional burden of inherited disorders and the visibility of conditions such as thalassemia and sickle-cell disease, which are tied to mandatory premarital testing programs. The Middle East carries one of the world’s highest prevalences of genetic disorders (approximately 20 per 1,000 live births, about double the global average), in part due to consanguinity patterns (2). However, awareness of “genetic diseases” does not necessarily translate into accurate understanding of prevention, risk, and service availability. In our data, about one-third had never encountered the concept of genetic counseling, consistent with reports that familiarity with genetic counseling remains limited in Arab communities (9). Importantly, a prior Palestinian national survey reported even lower awareness of the term “genetic counseling” (35.5%) (1), suggesting that our largely young and highly educated online sample may overestimate population-level knowledge.

Premarital screening testing (PMST) emerged as a critical theme. Attitudes were strongly favorable: agreement of PMST importance was associated with higher perception scores, and “maintaining the health of offspring” was the most endorsed motivator for genetic testing (97.2%). This aligns with other Middle Eastern findings that position PMST as a valued preventive tool (10). At the same time, knowledge about PMST limitations was incomplete. Only about 64% correctly understood that standard Palestinian PMST does *not* cover all genetic diseases, leaving over one-third with the mistaken belief that PMST essentially “guarantees” healthy offspring. Comparable misconceptions persist even in countries with long-established PMST programs, such as Saudi Arabia (8). This matters clinically and socially: if couples interpret a negative PMST as proof of “no genetic risk,” they may not seek counseling or additional testing, despite PMST typically focusing on a narrow panel (often hemoglobinopathies).

The TotalScore should be interpreted cautiously. It combines knowledge, perceived barriers, facilitators, and intention-related items. These are conceptually related to informed genetic decision-making but are not identical constructs. The modest internal consistency observed in this study supports the concern that the score is not a validated unidimensional measure of knowledge. For this reason, the revised analysis reports BarrierScore and MotivationScore separately, emphasizes item-level results, and treats the TotalScore as a descriptive composite index rather than a diagnostic category.

Social desirability bias is another important consideration. Several preventive-health responses showed very high endorsement, including the importance of PMST, willingness to seek counseling, and maintaining offspring health. In the Palestinian sociocultural context, protecting future children and supporting preventive screening may be perceived as socially expected answers. These reported intentions may not translate into real-world behavior. The contrast between high stated willingness to use counseling and the very low proportion who had actually visited a genetics clinic (8.8%) suggests a gap between hypothetical intentions and service utilization. This gap may reflect access barriers, limited referral pathways, cost, stigma, family pressures, or limited awareness of available services.

Encouragingly, respondents expressed strong receptivity to system-level solutions. Nearly 90% agreed that stronger government measures to reduce genetic disease would encourage genetic testing, and similarly high proportions supported community awareness campaigns and peer sharing of experiences. These views mirror trends in Saudi Arabia, where large majorities supported enhanced awareness and regulation (8). While our study did not directly measure whether Palestinians would proceed with marriage despite high-risk results, evidence from Yemen and Qatar suggests that a substantial proportion of couples may still marry even when PMST indicates genetic risks (10, 11). Therefore, improved knowledge is necessary but may be insufficient on its own; culturally sensitive counseling and supportive policies are likely needed to help at-risk couples translate information into risk-reducing actions.

Personal and family experience was a major driver of awareness. Participants reporting a family history of genetic disease scored higher and were independently more likely to be in the “Good” perception category (adjusted OR ~1.8). Those uncertain about family history had the lowest scores, suggesting that lack of exposure may contribute to lower perceived relevance or a false sense of security. This pattern aligns with evidence from Qatar showing that having affected relatives can significantly shape perceptions and receptivity toward genetic counseling and testing (12). Prior exposure may reduce psychological barriers and stigma-related concerns, consistent with literature indicating that familiarity with genetic services often lowers perceived barriers and increases comfort with testing (13). From a public health perspective, these findings support peer-led strategies: testimonials and community narratives may help close the “experience gap,” particularly for individuals without known family history who may underestimate genetic risks.

Education was the clearest sociodemographic predictor. University-educated participants had significantly higher awareness and perception scores than those with only high school education (moderate effect size, d ≈ 0.38). This echoes Palestinian and regional evidence linking education to improved genetic literacy (10, 11). Accordingly, less-educated groups represent a key target for tailored awareness campaigns. In contrast, overall composite scores did not differ significantly by sex (mean ~11.3 vs. 11.4, *p* = 0.44), which may reflect our sample’s homogeneously high education level and possible self-selection into a health-oriented online survey. However, item-level differences suggested that motivations and concerns may still vary by sex (discussed below). Age showed limited influence: younger adults had slightly higher scores than older groups, and the multivariable model suggested a modest decrease among those aged 36–45 compared with the 22–26 reference group, consistent with prior Palestinian findings that younger individuals can be more knowledgeable about genetic testing (1). Marital status and income were not significant predictors in this dataset, potentially due to limited variability (most reported middle income) and because undergoing mandated premarital testing may not automatically increase broader genetic literacy without counseling or active learning. These patterns should be interpreted cautiously given sample bias; national patterns may show larger gaps among rural, older, and lower-income communities that were underrepresented in our survey.

Item-level analysis highlighted specific strengths and gaps. Most respondents correctly rejected the false belief that genetic diseases “will not affect my children” (over 78% disagreed), indicating recognition that hereditary risk can affect any family. Yet only 58.8% reported knowing whether they personally have a genetic disease, implying substantial uncertainty about individual genetic status, reflecting limited access to screening beyond routine PMST. Awareness of genetic services was moderate: about two-thirds knew the role of genetic counseling clinics (63.3%) and had information about genetic testing (66.8%). Stigma-related concerns persisted for a sizable minority: around 36% endorsed fear about societal perceptions if diagnosed with a genetic disease.

Motivator items showed very strong endorsement overall, particularly child health protection (97.2%). Agreement that genetic testing enables reproductive options (including IVF with preimplantation genetic diagnosis) was also high, though regional variability existed. The least endorsed motivator related to restrictive marriage customs (55.7%), possibly reflecting that many did not feel bound by these traditions or did not believe testing would alter such customs. Sex differences appeared in select motivators: women more often endorsed government initiatives and hearing others’ experiences as encouraging factors, while men more often claimed they knew their own genetic status. Regional differences were also observed in certain areas (e.g., responsiveness to awareness campaigns), suggesting that local culture, service visibility, or community trust may shape how interventions are received. These granular patterns can inform targeted messaging addressing stigma where it is more prominent and strengthening outreach in regions less responsive to campaigns.

A large-scale Gaza study reported consanguinity rates around 40% in the current generation (with only a slight decline) and emphasized the need for further counseling and awareness (14). Overall, despite methodological differences across studies, the convergence of themes knowledge gaps about counseling, misconceptions about PMST, and strong support for awareness initiatives suggests these challenges are widespread across the region.

The findings have practical implications for public health practice in Palestine. First, PMST-related education should clearly explain what conditions are included and what limitations remain after a negative result. Second, genetic counseling should be integrated more visibly into premarital, maternal, child-health, and primary-care services. Third, awareness campaigns should be tailored to groups likely underrepresented in this survey, including rural communities, older adults, less-educated individuals, and people with limited digital access. Finally, stigma-sensitive framing is essential: counseling should be presented as a proactive family-health service rather than as a marker of social or marital unsuitability.

These findings point to clear priorities for Palestine: (1) strengthen education on genetic counseling and PMST limitations, and (2) improve access to counseling services. The misconception that PMST “guarantees” healthy offspring indicates the need for explicit messaging during premarital processes and through public campaigns. Since nearly 90% endorsed the idea that government measures and awareness campaigns would encourage testing, official public health messaging appears likely to be well received. Service expansion is also essential. Despite moderate awareness, utilization of genetics clinics was very low (8.8%), suggesting limited access, referral, or availability. Integrating genetic counseling into premarital services and maternal/child health settings supported by training for frontline healthcare providers could normalize counseling and improve public familiarity over time (15).

Given persistent stigma concerns among a minority, culturally sensitive approaches are needed, potentially involving community leaders and framing genetic counseling as a proactive family-health responsibility rather than a threat to social standing. Finally, outreach must include underserved communities that are likely underrepresented in web-based samples (rural areas, older adults, lower socioeconomic groups, and communities with higher consanguinity), to avoid widening health literacy gaps (16).

Strengths of this study include its focus on an underexplored public health topic in Palestine, inclusion of respondents from multiple regions, use of an adapted published questionnaire, and analysis of both composite scores and item-level patterns. The revised analysis also improves transparency by excluding under-18 submissions, reporting local reliability estimates, and clarifying the descriptive nature of TotalScore categories.

Limitations are substantial. The non-random, web-based convenience/snowball sampling strategy limits generalizability and likely over-represents younger, female, educated, urban-connected, and health-interested respondents. Because the survey link was openly distributed and forwarded, the number of individuals who received or viewed the invitation was unknown, so a response rate could not be calculated. The cross-sectional design prevents causal inference. Self-reported knowledge and attitudes may be affected by recall error, social desirability bias, and hypothetical-intention bias. The questionnaire was culturally adapted but was not formally pilot-tested in a separate Palestinian pilot sample, and internal consistency of the composite score was modest. Some subgroup analyses had small cell counts, and multiple bivariate comparisons increase the possibility of chance findings.

Future research should use probability-based or stratified sampling, include offline recruitment to reach digitally marginalized groups, and incorporate qualitative methods to explore stigma, family decision-making, service access, and reasons for the gap between stated willingness and actual counseling use. Intervention studies should test whether targeted education about PMST limitations and counseling pathways improves informed decision-making and service uptake.

## Conclusion

Among Palestinian adults in this web-based convenience sample, general awareness of genetic diseases was high, but awareness and use of genetic counseling were incomplete. Participants with family history of genetic disease, correct knowledge of disease onset, awareness of genetic counseling, awareness of PMST limitations, and stronger agreement with PMST importance showed more favorable TotalScores. Because the sample was not representative, the findings should not be generalized to the entire Palestinian adult population. The results support focused public health actions that explain PMST limitations, normalize genetic counseling, and improve access to culturally sensitive counseling services in premarital and primary-care settings.

## Data Availability

The raw data supporting the conclusions of this article will be made available by the authors, without undue reservation.
